# Treatment of hypocalcemia in hungry bone syndrome: A case report

**DOI:** 10.1016/j.ijscr.2018.08.011

**Published:** 2018-08-13

**Authors:** Farahnaz Anwar, Joseph Abraham, Ahmad Nakshabandi, Eugene Lee

**Affiliations:** Department of Internal Medicine, Mercy Hospital and Medical Center, Chicago, United States

**Keywords:** Hungry bone syndrome case report, Parathyroid hormone, Primary/secondary hyperparathyroidism, Hypocalcemia, Parathyroidectomy, Calcium gluconate/citrate, Calcitriol, Elemental calcium

## Abstract

**Introduction:**

Hungry bone syndrome (HBS) is rapid, intense and prolonged hypocalcemia that follows parathyroidectomy. The focus of this paper is HBS in patients with secondary hyperparathyroidism (SHPT) due to end stage renal disease (ESRD). Various risk factors are correlated with developing HBS post-parathyroidectomy due to SHPT which include: old age (>60 years); the preoperative level of parathyroid hormone (PTH); increased osteoclasts; and evidence of bone disease before surgery.

**Presentation of case:**

A 25-year-old woman, who underwent parathyroidectomy of all four parathyroid glands due to SHPT caused by ESRD. Her calcium deficit was prolonged, as expected in patients who undergo parathyroidectomy, however her calcium levels remained low despite unprecedented supplementation of elemental calcium and calcitriol.

**Discussion:**

Unfortunately, there is not enough data-based evidence to help prevent or minimize severe complications of hypocalcemia prior to parathyroidectomy. The main goal of treatment is replenishing the calcium deficiency through supplementation with calcium salts, high doses of active metabolites of vitamin D, and electrolytes.

**Conclusion:**

The ultimate goal of reviewing and analyzing this particular case is to obtain a better understanding for the treatment of Hungry bone syndrome. Although, there are very few cases as severe as this patient, hopefully this case study will result in greater insight and lead to improvement in the overall treatment of hypocalcemia.

## Highlights

• Patients with secondary hyper-parathyroidism experience severe and prolonged hypocalcemia following parathyroidectomy.
• Pre/postoperative management for HBS helps to minimize long-term effects.
• No clear set outline for management of HBS, treatment is on case-by-case bases.

## 1 Introduction

HBS is considered in patients following parathyroidectomy when the hypocalcemia is prolonged and severe, specifically total serum calcium less than 8.4 mg/dL (2.1 mmol/L) or ionized calcium less than 4.48 mg/dL (1.12 mmol/L) for more than four days post-surgery. It is believed to be caused due to the sudden drop in PTH levels and the impact that the hormone had on osteoclastic resorption [1]. The decrease in PTH is a result of unopposed activation of osteoblasts and increased influx of calcium into “calcium-starved” bone. HBS is reported most often after surgery for parathyroidectomy or thyroidectomy because of primary or secondary hyperparathyroidism. The longer the duration of elevated levels of PTH in the system, the greater the severity of hypocalcemia post parathyroidectomy.

There are few reported cases of HBS in literature, and data regarding incidence is sparse and varied. Patients who undergo parathyroidectomy due to primary hyperparathyroidism are believed to experience a milder form of the syndrome in comparison to patients with SHPT who develop a more severe and persist HBS. The incidence of the syndrome due to primary hyperparathyroidism (PHPT) ranges from 13 to 20%, while it can range from 27 to 51% for patients with SHPT [2]. Another review done in 2012 reported HBS to be found in 4 to 87% of patients who underwent parathyroidectomy [3].

The sudden decline of PTH causes an increase in bone density, and the longer the syndrome persists, the greater the increase in bone density. Some risk factors for developing HBS include: increased age; elevated levels of PTH preoperatively; increased number of osteoclasts on bone biopsy; as well as radiological evidence of bone disease prior to surgery [4].

Some clinical manifestations of severe hypocalcemia include perioral paresthesia, positive Chvostek and Trousseau signs, convulsions, carpopedal spasms, laryngospasm, and cardiac arrhythmias. Generally, six to 12 g per day of elemental calcium is considered safe to administer to patients, along with 2–4 μg/day of calcitriol [5]. The case discussed below is HBS with severe hypocalcemia in a 25-year-old patient following parathyroidectomy due to secondary hyperparathyroidism, that required unprecedented amounts of calcium during her post-operative treatment. This case study is constructed in order to satisfy the scare criteria [6].

## 2 Case report

A 25-year-old woman presented to the ENT clinic on April 2017 with complaints of generalized weakness and difficulty walking due to progressive worsening of leg pain. The leg pain had started several months before and was initially mild at onset, however, now caused the patient significant pain. Her past medical history included Bartter’s syndrome (diagnosed at age two), ESRD (2014), secondary hyperparathyroidism, previous pulmonary embolism (2016) and anemia of chronic disease. She was currently taking iron, thiamine, zinc, vitamin c, mag-ox, aspirin, lovenox and KCl. On physical examination- height was 158 cm, weight was 80.5 kg, vital signs were within normal limits. No other significant findings were noted.

**Fig. 1:**
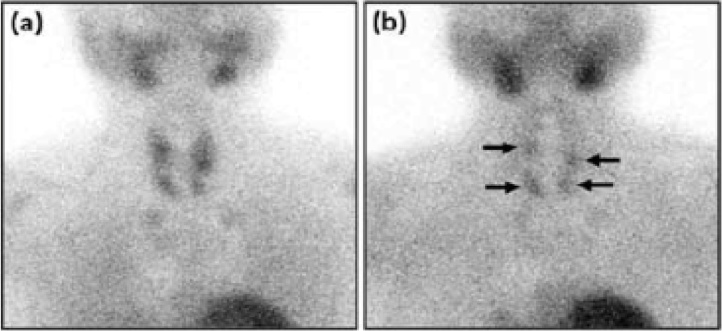
99mTc-sestamibi dual phase images showing a 4-gland parathyroid hyperplasia. (a) Tracer uptake by the thyroid and parathyroid glands in the early phase image. (b) Delated tracer washout from all the hyperplastic parathyroid glands (the arrows), in the late phase image.

The PTH level was 1849 pg/mL during the initial visit, therefore a Sestamibi scan was performed, however the scan did not show a localized adenoma. Fig. 1 shows a Sestamibi scan for a patient with secondary hyperparathyroidism.

The patient was immediately scheduled for resection of all four parathyroid glands based on several factors; including her PTH level, complaints of generalized weakness and worsening bone pain. The procedure was performed under general anesthesia, with no complications. Sections of the parathyroid glands were taken and sent for pathology. The PTH level intraoperatively was noted to be 71 pg/mL. The patient was then admitted to the ICU in stable condition following surgery. Goals that were set for the patient following surgery- 8.0–8.5 mg/dL in serum calcium and 4.5 mg/dL for ionized calcium.

On postoperative day (POD) one, the nephrologist was put on consult and became part of the patient’s care team till her discharge. They helped to determine daily amount of calcium administered and whether any adjustments were needed. Other recommendations made by the nephrologists: daily weights, hemodialysis twice per week, vitamin D supplements, and close observation of magnesium and phosphorus levels.

The development of hypocalcemia in the patient was rapid and progressive. On POD 1, the patient had a PTH level of 12 units/l, serum calcium level of 7.3 mg/dL and an ionized calcium of 2.9 mg/dL. However, she was asymptomatic. POD 2, the patient was stable and still in ICU, but complained of pain at the intravenous (IV) site on her right hand.

**Fig. 2:**
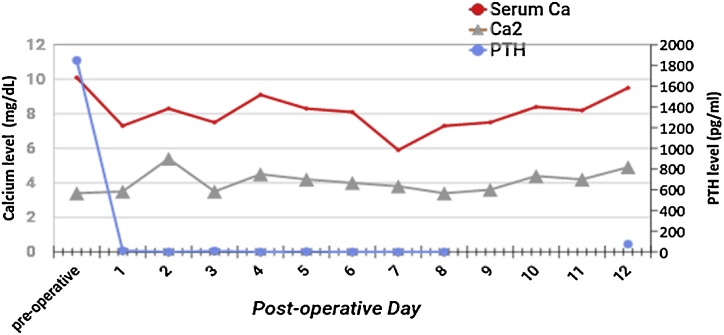
Changes in Serum Calcium and PTH postoperatively. Serum Ca: total serum calcium; Ca2: serum ionized calcium level; PTH: Parathyroid hormone.

She was scheduled for hemodialysis with high-calcium bath twice a week. The patient’s weight decreased 25 kg (from 80.5 kg to 55.4 kg) within a span of four days post-surgery. The baseline weight of the patient being unknown it can be assumed that the 80.5 kg was substantially higher than her norm and could potentially be attributed to the patient’s missed dialysis sessions as well as the bilateral lower extremity edema. Once the patient was able to tolerate oral calcium supplements she was transferred from the ICU to the internal floor. The patient received 11.37 g of elemental calcium, consisting of 27.5 g of calcium carbonate and 4 g of calcium gluconate, on her first day of oral calcium replacement. The concentrations of elemental calcium increased substantially thereafter, from 11.37 g to 17 g → 30 g → 35.9 g. The highest amount of calcium was provided on POD 11 where 28 500 (x6) g of calcium citrate was given to the patient, resulting in 35.9 g of elemental calcium. During the 11 days of treatment her total serum calcium maintained at an average of 7.8 mg/dL and ionized calcium at 4 mg/dL, while these levels are considered low they did not indicate the indicate to start the patient on intravenous calcium. Fig. 2 illustrates the patient’s changes in serum/ ionized calcium and PTH levels. The amount of calcium which this patient received has never been reported or recommended for any patient with HBS. The current recommendations are between 6–12 g/day [3]. The patients POD 7 laboratory values are presented in Table 1.

**Table 1:** Laboratory Results for Postoperative Day 7^a^.

Hematologic Labs		Routine Chemistry	
WBC	6.4 × 10(3)/mcL	Glucose	96 mg/dL
RBC	2.5 × 10(6)/mcL	Calcium level	7.4 mg/dL
Hemoglobin	6.4 gm/dL	Sodium	140 mmol/L
Hematocrit	20.30%	Potassium	4.3 mmol/L
MCV	81 fL	Chloride	102 mmol/L
Platelet	162 × 10(3) mcL	CO2	27 mmol/L
Iron	37 mcg/dL	Alk Phos	696.0 unit/L
TIBC	195 mcg/dL	BUN	21 mg/dL
Ferritin	535 ng/mL	Creatinine	4.51 mg/dL
Transferrin	130 mg/dL	BUN/Creatinine Ratio	5
		Albumin Level	2.7 gm/dL
		Total Protein	5.0 gm/dL

TIBC: Total Iron Binding Capacity, WBC: white blood cell count, BUN: blood urea nitrogen, Alk Phos: Alkaline phosphatase, RBC: Red blood cell count, MCV: Mean corpuscular volume.

a POD 7 is when the patient was transferred from the ICU to the internal team for care.

**Fig. 3:**
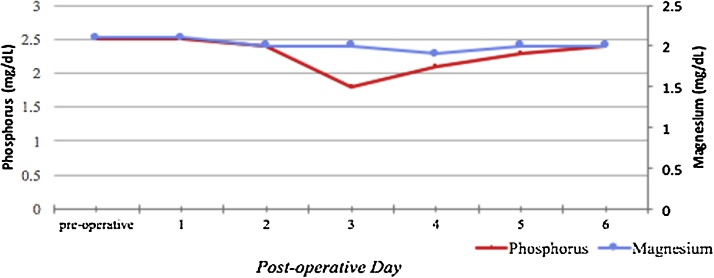
Changes in Electrolytes (Phosphorus and Magnesium) Postoperatively.

**Fig. 4:**
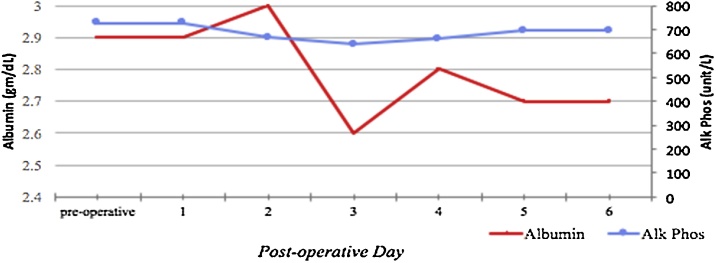
Albumin and Alkaline phosphatase levels postoperatively. *Alk Phos: Alkaline Phosphatase.

The treatment was stopped abruptly due to the patient’s request. She was discharged with 2 days of medication and given prescription for another 14 days. The importance of maintaining her bi-weekly dialysis appointments was discussed with the patient, where her serum calcium, ionized Ca, BMP and Alkaline phosphatase will be closely monitored. Calcium levels on discharge were - serum calcium of 9.5 mg/dL and ionized calcium of 4.9 mg/dL. Had the patient not requested discharge, the medical team wanted to continue the ongoing treatment plan. That plan involved providing the necessary amount of calcium, closely monitoring her serum levels as well as her weight for an additional few days (Fig. 3, Fig. 4).

The discharge medications included: calcium citrate 30 g/QID; calcitriol 5 mcg/daily; calcium citrate 200 mg/QID; epoetin alfa 10 000 units/mL. The oral calcium supplement (ca citrate) was prescribed to help increase and maintain the serum calcium levels. Epoetin alfa is a synthetic erythropoietin analog, which stimulates the bone marrow to produce more red blood cells and calcitriol was prescribed to function as an active form of Vitamin D.

## 3 Discussion

After parathyroidectomy hypocalcemia is expected however it usually resolves within 2-4-days post-surgery. If it persists for more than 4 days or drops below 2.1 mmol/L then the patient is diagnosed as having HBS.

Risk factors of developing hungry bone syndrome in patients with SHPT due to ESRD are: age 60 or above; serum alkaline phosphatase three times above the upper limit of normal preoperatively; PTH level greater 1000 pg/mL; increased number of osteoclasts on bone biopsy; radiological findings of lytic lesions; brown tumors, as well as periosteal erosions and fractures [1]. Although, the weight and volume of the resected parathyroid tissue is a risk factor for patients with primary hyperparathyroidism, it doesn’t increase the risk for SHPT patients in developing HBS. Older patients are believed to be more susceptible to developing HBS, due to lower levels of serum calcitriol which occurs with age and decreased 1-alpha-hydroxylase activity [7]. The patient discussed above had the following risk factors preoperatively: high levels of serum alkaline phosphatase (>727 unit/L) and elevated levels of PTH (1849 pg/mL) which could explain why she developed a severe form of HBS as well as her lack of response to the treatment. Since there was no radiologic testing done for the patient prior to surgery, confirming whether lytic lesions were also a risk factor is undetermined.

**Fig. 5:**
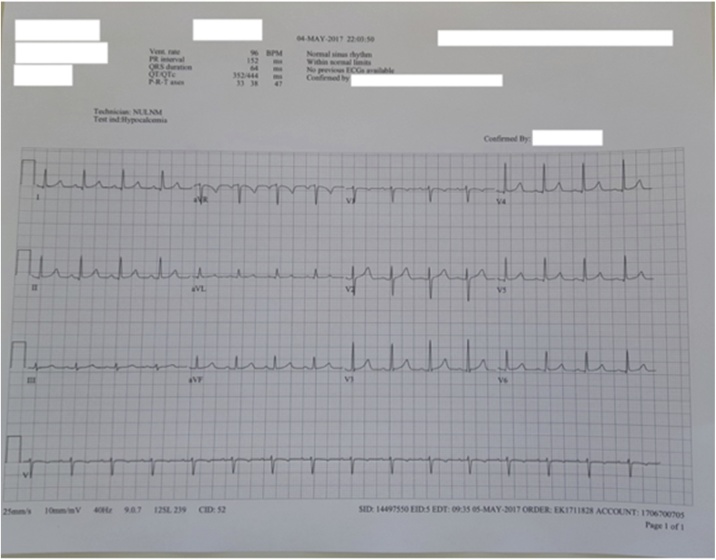
Electrocardiogram Post-surgery Post-parathyroidectomy.

Some of the common signs and symptoms attributed with hypocalcemia are acroparesthesias (perioral, fingers and toes), tetany, seizures, confusion as well as cardiac symptoms such as QT prolongation and arrhythmias. The neuromuscular symptoms become evident when serum ionized calcium is less than 4 mg/dL or total serum calcium is < 7.5 mg/dL [4]. Remarkably, even with much lower amounts of serum calcium, our patient did not exhibit any hypocalcemia symptoms. Rather, her EKG actually demonstrated a normal sinus rhythm (Fig. 5). Some of the symptoms she did experience during her recovery but which subsided before her discharge included: weakness, fatigue, nausea, bilateral lower leg non-pitting edema, as well as pain and swelling at the IV site on her right hand.

Subtotal parathyroidectomy (SPTX) and total parathyroidectomy with auto-transplantation (TPTX + AT) are options beside total parathyroidectomy for patients who require surgery for hyperparathyroidism. Although, SPTX and TPTX + AT can be considered as standard of care for secondary hyperthyroidism, they have been linked to recurrence rates of 10–12% in 3 years post-surgery. While, the recurrence rates for total parathyroidectomy without auto-transplantation are between 0–4% in the same 3-year follow-up with similar morbidity rates to SPTX and TPTX + AT [8].

Serum magnesium and phosphorus concentrations have also decreased post-surgery in patients with HBS. Hyperkalemia has been noted in patients diagnosed with having HBS and undergoing dialysis due to ESRD. Successful removal of hyperactive parathyroid tissue can be identified with a decrease in the level of serum PTH intraoperatively by more than 50% or below 300 pg/mL, especially in patients with ESRD. Prior to undergoing parathyroidectomy, this patient’s PTH level was 1849 pg/mL, and intraoperatively it was measured to be 71 pg/mL, indicating successful removal of the hyperactive glands. Serum alkaline phosphatase could increase immediately following surgery or remain at preoperative levels for weeks and normalize eventually in the months to come. The increase in alkaline phosphatase was also observed in our patient (727 unit/L) and remained above 600 unit/L for the entirety of her hospital recovery period. The patient had started hemodialysis bi-weekly since her diagnosis of ESRD in 2014 and the same schedule was maintained during her hospital admission.

Post-parathyroidectomy patients with PHPT are reported to have a greater increase in bone density in comparison to SHPT. The increase in bone density of the lumbar spine and greater trochanter has been studied in patients in several case reports, and the following was observed: 17% increase in bone density at 10 weeks, 10% at 6 months, and 27–65% at the one-year post-surgery for the lumbar spine. In terms of the greater trochanter, the following was recorded of the change in bone density: 33% at six months, and 35–131% at one year after parathyroidectomy [3]. In another study, patients with SHPT post-parathyroidectomy were noted to have an increase in bone density at post-six months, however, some patients were observed to have osteomalacia [9]. A decrease in the number of lytic lesions, brown tumors and fracture sites was noted at one-year post-parathyroidectomy in the radiographic findings of patients. A skeletal scintigram done one-month post-surgery in a case report in 2010 for HBS, showed an increase in radioactive isotope uptake, known as “flare phenomenon” [10]. This phenomenon is believed to be the body’s response to the rapid increase in bone formation, especially the increase in calcium uptake. Bone scintigraphy demonstrating this phenomenon is rarely ordered and thus incidence rates are believed to be underreported. As no bone scintigram was performed for this patient, this case study likely contributes to the phenomenon’s underreporting.

The recommended treatment for hungry bone syndrome involves administration of elemental calcium, between 6–12 g/day. Calcium is initially administered intravenously and switched to oral supplements when the patient can swallow with any discomfort. Calcium salts (citrate, gluconate, carbonate) are preferred due to their high elemental calcium availability. Calcium carbonate is composed of 40.0 percent elemental calcium and is released during digestion, while calcium citrate is composed of 21.1 percent, and calcium gluconate contains 9.3 percent. Two-four mg/day of calcitriol in the preoperative and postoperative periods is also highly recommended and has shown to help maintain serum calcium levels post-surgery. The calcium requirement for patients with SHPT post-parathyroidectomy is 3.2 g at week one and gradually decreasing to about 2.4 g at week six [11]. Based on a study from Thailand, reduced concentrations of calcium were recommended for patients with SHPT who were initially started on high dose (2.25–4 mg/day) rather than the low-dose of 0.75–1.5 mg/day of calcitriol. Calcium drip at 1 mg/kg/h is generally used immediately after surgery and titrated down once patient receives 100–200 mg of elemental calcium.

The 25-year patient discussed in this report was administered 11.37 g of elemental calcium on her POD one and the amount continuously increased to a total of 35.9 g by the end of the second week post-surgery. As mentioned, she did have a few risk factors that may have contributed to her refractory hypocalcemia, however, there has been no report yet in literature of a case that required such unprecedented amounts of calcium especially for this duration of time.

## 4 Conclusion

Although the exact incidence rates of HBS is not well researched, hypocalcemia is believed to occur quite often following parathyroidectomy in patients with PHPT and SHPT. SHPT patients who undergo surgery generally experience a more severe and prolonged course in comparison to patients with PHPT. As of 2017, there are no clear outlines for the management of HBS; treatment for each patient is different in terms of dose and frequency. Regardless of the ill-defined or non-existent treatment outline for the syndrome, there has yet to be a case published claiming to have used these concentrations of elemental calcium. More studies in pre/postoperative management for HBS need to be conducted to avoid severe hypocalcemia and ultimately minimize any long-term effects, such as those discussed for the patient above.

## Conflicts of interest

N/A.

## Funding

N/A.

## Ethical approval

This case study did not require ethical approval, as per the guidelines of my medical school (Saint James School of Medicine). This case study was written to simply outline the severity of the patient’s condition and its management. No new treatment or test was conducted in order to achieve the desired results.

## Consent

Consent was obtained from the patient, regarding publishing of the information.

## Author contribution

Dr. Anwar – study design, data collection, analysis and interpretation, drafting and writing the paper.

Dr. Abraham – provided explanation for laboratory findings/ trends observed during post-operative period and treatment of patient.

Dr. Nakshabandi – helped with research, data interpretation and editing.

Dr. Lee – part of the medical team.

## Registration of research studies

UIN: researchregistry3764.

## Guarantor

Dr. Farah Anwar.
